# The BH3‐only protein NOXA is essential for apoptosis induction by BH3‐mimetics targeting BCL2 or BCL‐X_L_ in DLBCL

**DOI:** 10.1111/bjh.70192

**Published:** 2025-10-05

**Authors:** Nahide Yildirim, Marius Anders, Victoria M. Smith, Sandrine Jayne, Moritz Assmann, Rebekah Jukes‐Jones, Martin J. S. Dyer, Meike Vogler

**Affiliations:** ^1^ Institute for Experimental Pediatric Hematology and Oncology, Goethe University Frankfurt Frankfurt Germany; ^2^ The Ernest and Helen Scott Haematological Research Institute Leicester Cancer Research Centre, University of Leicester Leicester UK; ^3^ German Cancer Consortium (DKTK) Partner Site Frankfurt/Mainz, a Partnership Between DKFZ and University Hospital Frankfurt Frankfurt Germany; ^4^ University Cancer Center Frankfurt (UCT), University Hospital Frankfurt, Goethe‐University Frankfurt Frankfurt Germany

**Keywords:** apoptosis, BCL2 proteins, BH3‐mimetics, DLBCL, venetoclax

## Abstract

BCL2 inhibitors (BCL2i) have transformed the management of chronic lymphocytic leukaemia (CLL), but their use in more aggressive B‐cell malignancies such as diffuse large B‐Cell lymphoma (DLBCL) is complicated by the more heterogeneous nature of the disease. Successful responses are limited to a subset of patients, highlighting the need for robust biomarkers predicting sensitivity. Here, we investigated the underlying mechanisms of inherent resistance to the BCL2i ABT‐199 and BCL‐X_L_ inhibitor A1331852, focusing on the roles of the principal pro‐apoptotic BH3‐only proteins NOXA and BIM. We show that NOXA deletion, but not BIM deletion, in BCL2 and BCL‐X_L_‐dependent DLBCL cells both in vitro and in vivo resulted in a highly significant enhanced resistance to both BCL2i and BCL‐X_L_ inhibitors. In contrast, NOXA deletion did not result in alteration of sensitivity to MCL1 inhibitors. NOXA loss was associated with increased stability and binding capacity of MCL1; binding of BIM to MCL1 was associated with resistance to ABT‐199. Resistance to BCL2i and BCL‐X_L_ inhibitors was abrogated by suppression of MCL1 expression. In conclusion, we show that NOXA is essential for the effectiveness of BH3‐mimetics targeting BCL2/BCL‐X_L_; in the absence of NOXA, BIM displaced from BCL2/BCL‐X_L_ can be bound by MCL1.

## INTRODUCTION

The induction of apoptosis is critically regulated by the B‐cell lymphoma 2 (BCL2) protein family. Consequently, the anti‐apoptotic BCL2 proteins are frequently overexpressed in cancer. High expression of BCL2 or related anti‐apoptotic proteins, such as BCL‐X_L_, MCL1 or BCL2A1, prevents the activation of the pro‐apoptotic BCL2 proteins BAX and BAK that permeabilize the outer mitochondrial membrane to facilitate the release of cytochrome c into the cytosol. To induce cytochrome c release, BAX or BAK oligomerizes within the outer mitochondrial membrane^1^ which is prevented by the anti‐apoptotic BCL2 proteins in different ways: first, they can directly bind and sequester BAX/BAK to prevent their oligomerization, and second, they can sequester and inhibit the pro‐apoptotic BH3‐only proteins. BH3‐only proteins are activated upon cellular stress, leading to their accumulation at mitochondrial membranes. Here, BH3‐only proteins can either counteract the anti‐apoptotic BCL2 proteins or directly facilitate BAX/BAK oligomerization.^2^ Some BH3‐only proteins like BIM may be able to promiscuously inhibit all anti‐apoptotic BCL2 proteins and simultaneously trigger direct BAX/BAK activation, while other BH3‐only proteins like NOXA display a more limited binding profile and only bind to selected partners. Thus, the BCL2 protein family resembles a network of pro‐ and anti‐apoptotic factors that determines cell fate in a highly cell type‐ and situation‐dependent manner.

Given their ability to tip the scales towards apoptosis induction, functionally mimicking the BH3‐only proteins offers a promising strategy for the treatment of cancer.^3^ Small molecule BH3‐mimetics have been developed that either selectively target individual anti‐apoptotic BCL2 proteins or subsets with shared homology within their hydrophobic grooves.^4^, ^5^ The discovery of ABT‐199 (venetoclax/venclyxto) as a selective BCL2i has enabled the clinical translation of this mechanistic insight, and ABT‐199 has now transformed the treatment of some types of leukaemia.^5^, ^6^, ^7^

However, clinical responses in other malignancies including diffuse large B‐Cell lymphoma (DLBCL) were disappointing, and early phase I trials showed limited activity of ABT‐199 as a single agent with only 18% of DLBCL responding to treatment.^8^ Preclinical studies have highlighted that, in line with their highly heterogeneous nature,^9^, ^10^, ^11^ DLBCL cells exhibit a wide range of BCL2 family protein expression. BCL2, BCL‐X_L_ and MCL1 are abundantly expressed independently of the DLBCL histological subtype, usually in combination.^12^, ^13^ Apart from BCL2, BCL‐X_L_ and MCL1 may also represent promising therapeutic targets with a subset of DLBCL cells displaying high sensitivity to BH3‐mimetics selectively targeting BCL‐X_L_ (e.g. A1331852) or MCL1 (e.g. S63845).^13^ Although direct inhibition of MCL1 may be associated with toxicities preventing clinical applications,^14^ the heterogeneous response to BH3‐mimetics observed in DLBCL necessitates the discovery of resistance mechanisms to BCL2i and predictive biomarkers to inform and guide clinical development.

Here, we investigated the response to BH3‐mimetics in a selected panel of DLBCL cell lines and focused on the role of the BH3‐only proteins NOXA and BIM. Importantly, previous genome‐wide genetic screens performed in DLBCL cell lines sensitive to ABT‐199 indicated that NOXA rather than BIM was an essential mediator of apoptosis induced by ABT‐199.^15^, ^16^, ^17^ Comparably, deletion of NOXA induced ABT‐199 resistance in AML cells, while genetic amplification of the gene encoding for NOXA conferred sensitivity to ABT‐199.^18^ Given that NOXA selectively interacts with MCL1 and potentially BCL2A1, but not with BCL2 or BCL‐X_L_, these data point to an important crosstalk between BCL2 and MCL1 which is required for ABT‐199 to induce apoptosis. In this study, we genetically deleted NOXA in DLBCL cells that were sensitive either to ABT‐199 or to A1331852 and investigated the interaction between pro‐ and anti‐apoptotic BCL2 proteins. Our data highlight that, in the absence of NOXA, displaced BIM is sequestered by MCL1 to block apoptotic signalling.

## METHODS

### In vivo experiments

Animal experiments conformed to the British Home Office Regulations (Animal Scientific Procedures Act 1986; Project Licences P8E5F4055 and PP9907621) and the guidelines for the welfare and use of animals in cancer research.^19^ This work was locally approved by the University of Leicester Animal Welfare Ethical Review Body subcommittee.

Additional details on methods can be found in the Supporting Information.

## RESULTS

### Limited response to ABT‐199 despite high BCL2 expression

**FIGURE 1:**
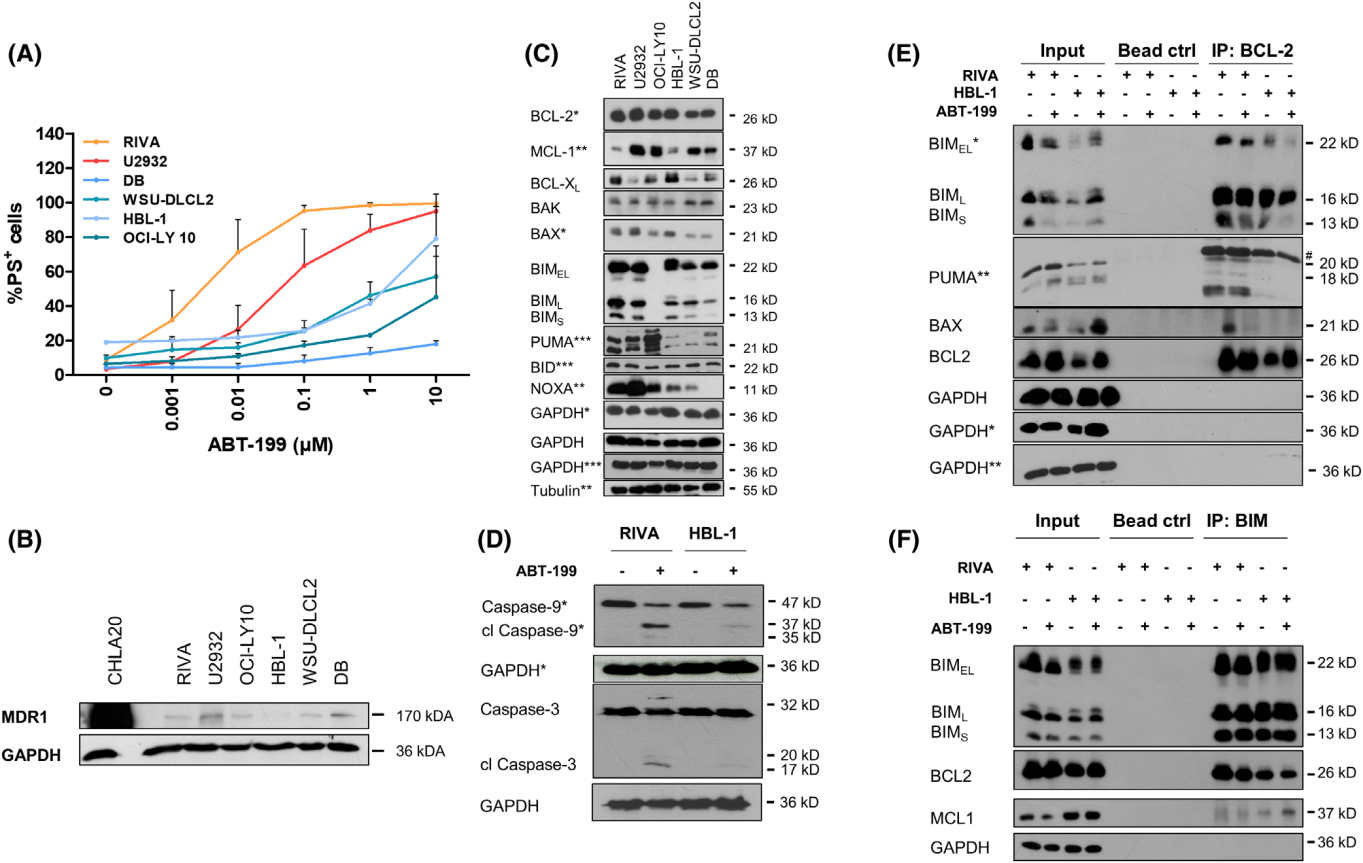
Heterogeneous response to ABT‐199 in DLBCL. (A) DLBCL cell lines were exposed to different concentrations of ABT‐199 for 24 h before analysis of apoptosis using AnnexinV‐FITC and flow cytometry. Data presented are mean + SD (*n* > 3). (B) Expression of MDR1 was analysed by western blotting with cisplatin‐resistant CHLA20 neuroblastoma cells serving as positive control. (C) Expression of BCL2 proteins was assessed in the different cell lines by western blotting with GAPDH and Tubulin serving as loading controls. Representative experiment of *n* = 4 is shown with symbols (*, **) representing individual experiments. (D) RIVA or HBL‐1 cells were treated with ABT‐199 (0.3 μM) for 6 h before analysis of caspase cleavage by western blotting. Representative experiment of *n* = 2 is shown with GAPDH served as loading control. (E, F) RIVA or HBL‐1 cells were exposed to ABT‐199 (0.1 μM) in the presence of zVAD.fmk (20 μM) for 6 h before lysis and immunoprecipitation of BCL2 (E) or BIM (F). (E) Binding of BIM, PUMA and BAX was assessed by western blotting with GAPDH serving as loading control. Representative experiment of *n* = 3 is shown with symbols (*) indicating different blots of the same experiment and # indicating an unspecific band. (F) Binding of BCL2 and MCL1 was assessed by western blotting with GAPDH serving as loading control. Representative experiment of *n* = 2 is shown.

To investigate patterns associated with the response to ABT‐199, a selection of DLBCL cell lines was exposed to ABT‐199. In line with previous reports,^13^, ^20^ DLBCL cells displayed a heterogeneous response with some cell lines (RIVA, U2932) displaying sensitivity to nanomolar concentrations of ABT‐199 (Figure 1A; Figure S1A). To investigate whether the response was associated with the expression of the drug efflux transporter ABCB1/MDR1,^21^ we assessed the expression of MDR1 but found only limited expression in DLBCL, as compared to a positive control cell line (Figure 1B). Although BCL2 levels were higher in the sensitive RIVA and U2932 cells, BCL2 was also abundantly expressed in the more resistant cell lines (Figure 1C). The related anti‐apoptotic BCL2 proteins, BCL‐X_L_ and MCL1, were not differentially expressed in the more resistant cells. In a selected sensitive (RIVA) and a resistant cell line (HBL‐1), the response to ABT‐199 was associated with activation of the intrinsic apoptotic pathway, as assessed by cleavage of caspase‐9 and caspase‐3 (Figure 1D).

Besides the expression of the BCL2 family proteins, sensitivity to BH3‐mimetics may be influenced by the priming status of the cells, which is indicated by the interaction between pro‐ and anti‐apoptotic BCL2 family members. To investigate the binding partners of BCL2 and the effect of ABT‐199 on the binding partners, immunoprecipitation (IP) experiments were performed (Figure 1E,F). In both sensitive RIVA and resistant HBL‐1 cells, the BH3‐only proteins BIM and PUMA were bound to BCL2, with less PUMA being expressed and bound to BCL2 in the HBL‐1 cells. Upon ABT‐199 treatment, a proportion of BIM_S_ was displaced from the target protein BCL2. Interestingly, in the more resistant HBL‐1 cells, ABT‐199 treatment induced some BIM binding to MCL1 (Figure 1F), indicating that MCL1 may play a role in the sequestration of pro‐apoptotic binding partners. Additionally, an interaction was observed between BCL2 and the multidomain pore‐forming protein BAX in the sensitive RIVA but not the HBL‐1 cells, which was displaced by ABT‐199 (Figure 1E).

### NOXA is required for ABT‐199‐induced apoptosis

**FIGURE 2:**
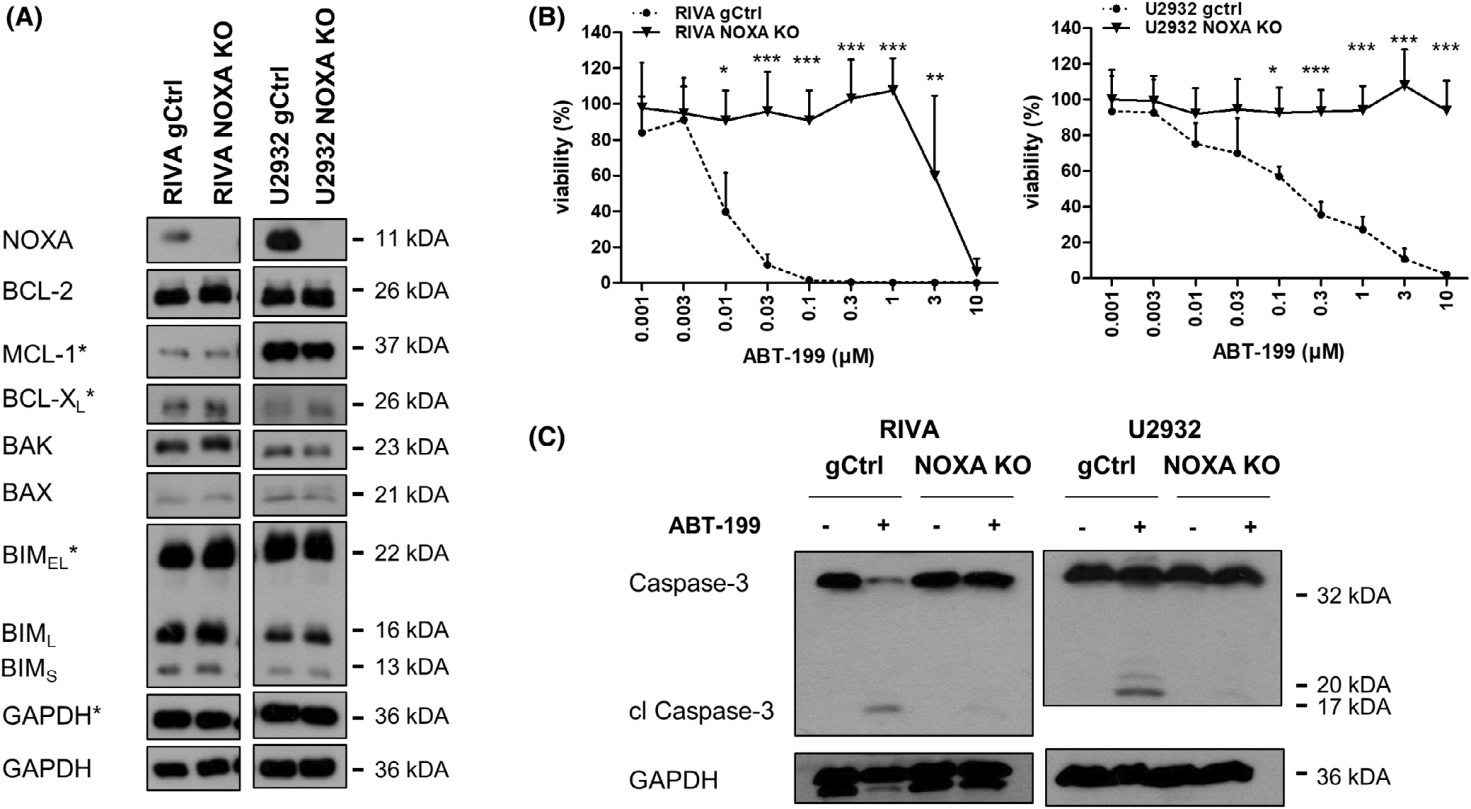
Deletion of NOXA confers resistance to ABT‐199. (A) KO of NOXA was performed in RIVA and U2932 cells using CRISPR/Cas9. KO of NOXA and expression of BCL2 proteins in control (gCtrl) and NOXA KO cells was assessed by western blotting with GAPDH serving as loading controls and symbols (*) indicating individual blots required to detect all proteins. (B) Control and NOXA KO cells were exposed to different concentrations of ABT‐199 for 72 h before analysis of cell viability using CTG assay. Data presented are mean + SD (*n* > 3). *p < 0.05; **p < 0.01; ***p < 0.001. (C) Control and NOXA KO cells were exposed to ABT‐199 (0.1 μM) for 6 h before analysis of caspase cleavage using western blotting with GAPDH serving as loading control.

The sensitive cell lines RIVA and U2932 also displayed high expression of NOXA, while the more resistant cells display low or absent NOXA expression (Figure 1C). To investigate further the role of NOXA in ABT‐199‐induced cell death, we performed CRISPR/Cas9‐mediated knockout (KO) of NOXA in the ABT‐199‐sensitive RIVA and U2932 cells (Figure 2A). Loss of NOXA resulted in a dramatically reduced sensitivity to ABT‐199, with both RIVA and U2932 cells tolerating ~1000‐fold higher ABT‐199 concentrations (Figure 2B), which was confirmed by the absence of caspase‐3 cleavage (Figure 2C). To investigate whether a similar effect could be observed for the activator‐type BH3‐only protein BIM, we performed stable KO of BIM in the same cells. Interestingly, deletion of BIM had no significant effect on ABT‐199 sensitivity (Figure S1B,C), highlighting that NOXA may have unique functions that are essential for ABT‐199‐induced apoptosis.

These findings also imply that increasing NOXA levels in cells with low endogenous NOXA may increase the efficacy of ABT‐199. To test this hypothesis, we selected a range of compounds described to increase NOXA expression, including the histone deacetylase inhibitor panobinostat,^16^ the proteasome inhibitor bortezomib,^22^ the bromodomain and extraterminal protein inhibitor dBET6^23^ and the neddylation inhibitor MLN4924.^24^ Surprisingly, NOXA protein levels were only upregulated by bortezomib, which also increased ABT‐199‐induced cell death in line with previous reports (Figure S2).^25^, ^26^

**FIGURE 3:**
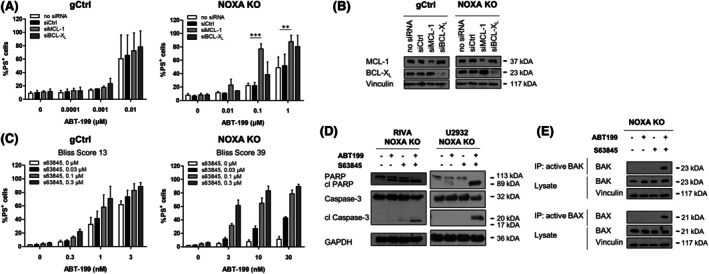
MCL1 is important for the function of NOXA. (A) Transient silencing of MCL1 or BCL‐X_L_ was performed in RIVA control (gCtrl) or NOXA KO cells before treatment of cells with different concentrations of ABT‐199 for 24 h and analysis of apoptosis using AnnexinV‐FITC and flow cytometry. Non‐transfected (no siRNA) or non‐targeting siRNA (siCtrl) were used as controls. Data presented are mean + SD (*n* > 3). **p < 0.01; ***p < 0.001. (B) Knockdown efficacy was assessed by western blotting with Vinculin serving as loading control. (C) RIVA control (gCtrl) or NOXA KO cells were treated with a combination of ABT‐199 and S63845 for 24 h before analysis of apoptosis using AnnexinV‐FITC and flow cytometry. Data presented are mean + SD (*n* > 3). Bliss score is indicated for the respective treatment. (D) Cells were treated with ABT‐199 (0.01 μM) and S63845 (0.3 μM) for 24 h before analysis of PARP and caspase‐3 cleavage with GAPDH serving as loading control. Representative experiment is shown (*n* = 2). (E) Cells were treated with ABT‐199 (0.01 μM) and S63845 (0.3 μM) for 6 h before analysis of BAX and BAK activation using immunoprecipitation with antibodies against the active conformation of BAK and BAX. Lysate is shown for equal loading with Vinculin serving as loading control. Representative experiment is shown (*n* = 2).

Next, we asked how NOXA might influence the response to ABT‐199 on a molecular level. To investigate the role of its main binding partner MCL1, we performed siRNA‐mediated silencing of MCL1 in the gCtrl and NOXA KO cells. In the gCtrl cells, silencing of MCL1 had no effect on ABT‐199‐induced apoptosis. However, silencing of MCL1 significantly resensitized NOXA KO cells to ABT‐199‐induced apoptosis. In contrast, silencing of the related protein BCL‐X_L_ had no effect in either the gCtrl or the NOXA KO cells (Figure 3A,B). Similarly, treatment with the MCL1 inhibitor S63845 was able to increase ABT‐199‐induced cell death more synergistically in the NOXA KO cells than the gCtrl cells (Figure 3C). Synergistic induction of apoptosis by ABT‐199 and S63845 in the NOXA KO cells was confirmed by analysis of caspase cleavage (Figure 3D) as well as BAX and BAK conformational changes associated with their pore‐forming ability (Figure 3E). Taken together, these data suggest that, in the absence of NOXA, MCL1 becomes functionally more important and compensates for the inhibition of BCL2 by ABT‐199 to prevent apoptosis induction.

### Impact of NOXA on BCL2 protein interactions

**FIGURE 4:**
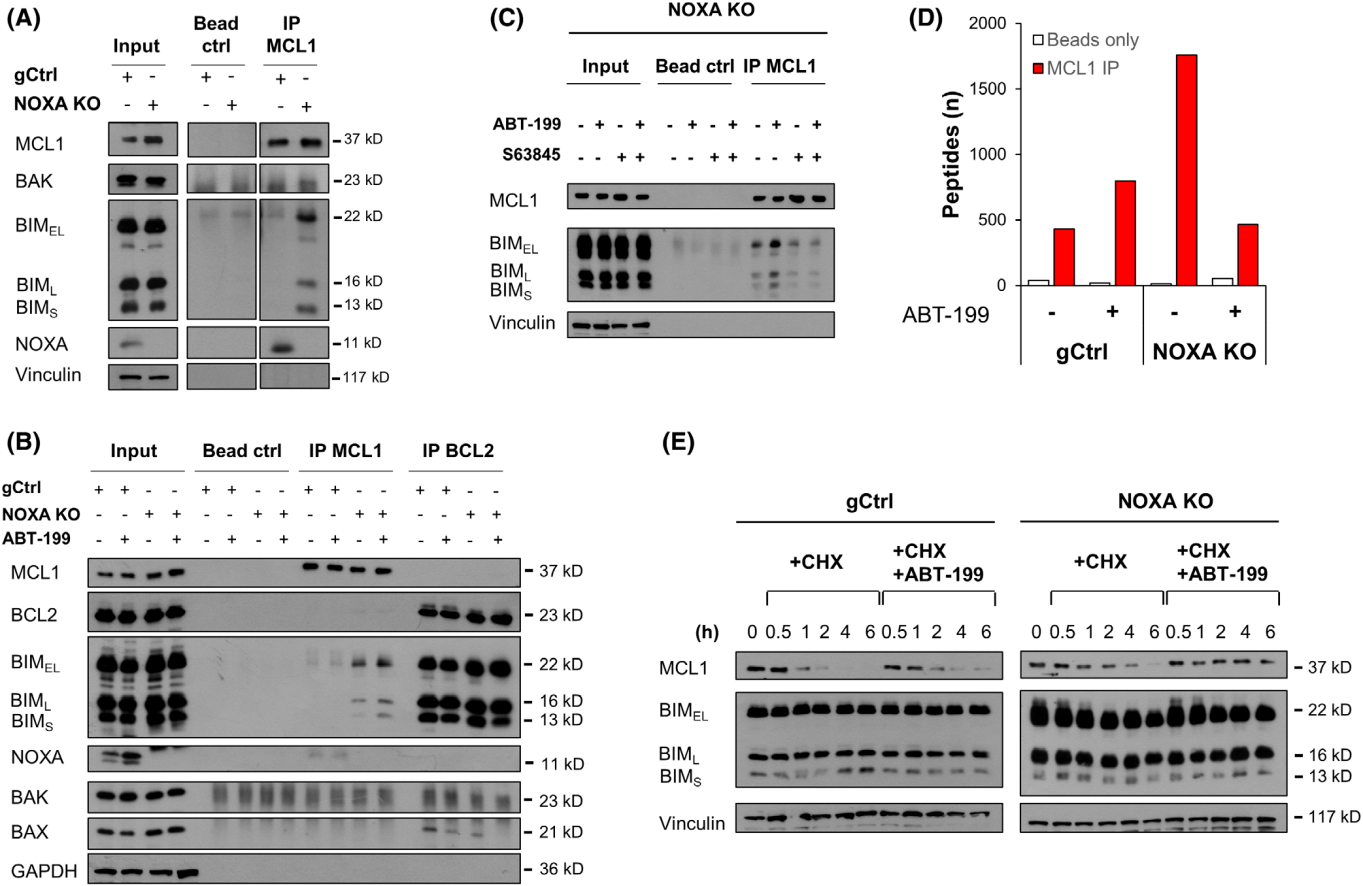
Altered interaction within BCL2 proteins leads to altered stability of MCL1. (A) Immunoprecipitation of MCL1 in RIVA control (gCtrl) or NOXA KO cells was analysed for binding of BAX, BIM and NOXA using western blotting. (B) RIVA control or NOXA KO cells were treated with ABT‐199 (0.1 μM) in the presence of zVAD.fmk (20 μM) for 6 h before immunoprecipitation of MCL1 or BCL2. Binding of BIM, NOXA, BAX, and BAK was assessed by western blotting with GAPDH serving as loading control. Representative experiment is shown (*n* = 2). (C) RIVA NOXA KO cells were treated with ABT‐199 (0.01 μM) and S63845 (0.3 μM) in the presence of zVAD.fmk (20 μM) for 6 h before immunoprecipitation of MCL1. Binding of BIM was assessed by western blotting with GAPDH serving as loading control. Representative experiment is shown (*n* = 2). (D) MS analysis of MCL1 IP and beads only control with peptide counts for the interacting proteins. (E) Protein stability was assessed in the RIVA gCtrl or NOXA KO cells by treatment with cycloheximide (CHX, 100 μg/mL) and ABT‐199 (1 μM) for up to 6 h. Western blotting was performed with Vinculin serving as loading control.

Deletion of NOXA may create free binding capacity and hence alter the MCL1 interactome. IP of MCL1 indeed showed enhanced binding of BIM to MCL1 upon NOXA deletion (Figure 4A). Inactivation of BCL2 by ABT‐199 resulted in increased binding of BIM to MCL1, indicating that NOXA may prevent the ability of MCL1 to sequester BIM upon its displacement from BCL2 (Figure 4B). Co‐treatment with ABT‐199 and S63845 resulted in displacement of BIM from MCL1 in the NOXA‐deleted cells, providing a potential explanation for the observed synergy between ABT‐199 and S63845 in the NOXA‐deleted cells (Figure 4C).

To further explore the free binding capacity of MCL1 in the absence of NOXA, we performed an IP of MCL1 followed by MS analysis. Of note, in the gCtrl cells, NOXA was only detected at very low peptide counts in the MCL1 IP, which may reflect its small size of only 11 kDa. In total, 129 proteins were found to potentially associate with MCL1 in the RIVA cells, with many of these being involved in mitochondrial metabolism or ribosomes. The greatest number of potential binding partners was detected in the untreated NOXA KO cells (Figure 4D; Figure S3), confirming that the absence of NOXA creates more MCL1‐binding capacity. To investigate whether any proteins displaced from BCL2 by ABT‐199 subsequently bound to MCL1, we compared untreated and ABT‐199 treated samples. As expected, upon treatment with ABT‐199, BIM (encoded by *BCL2L11*) bound to MCL1 increased. The only other protein that showed a similar tendency was VDAC3 (Figure S3). Binding of BIM or NOXA to MCL1 has previously been shown to influence the stability of MCL1 by regulating its proteasomal degradation. Cycloheximide chase experiments with or without treatment with ABT‐199 demonstrate that MCL1 is more stable and has a longer protein half‐life upon NOXA deletion, and that its stability is further increased by treatment with ABT‐199 and the associated binding of BIM to MCL1 (Figure 4E).

### NOXA is required for apoptosis induced upon BCL‐X_L_ inhibition but not upon MCL1 inhibition

**FIGURE 5:**
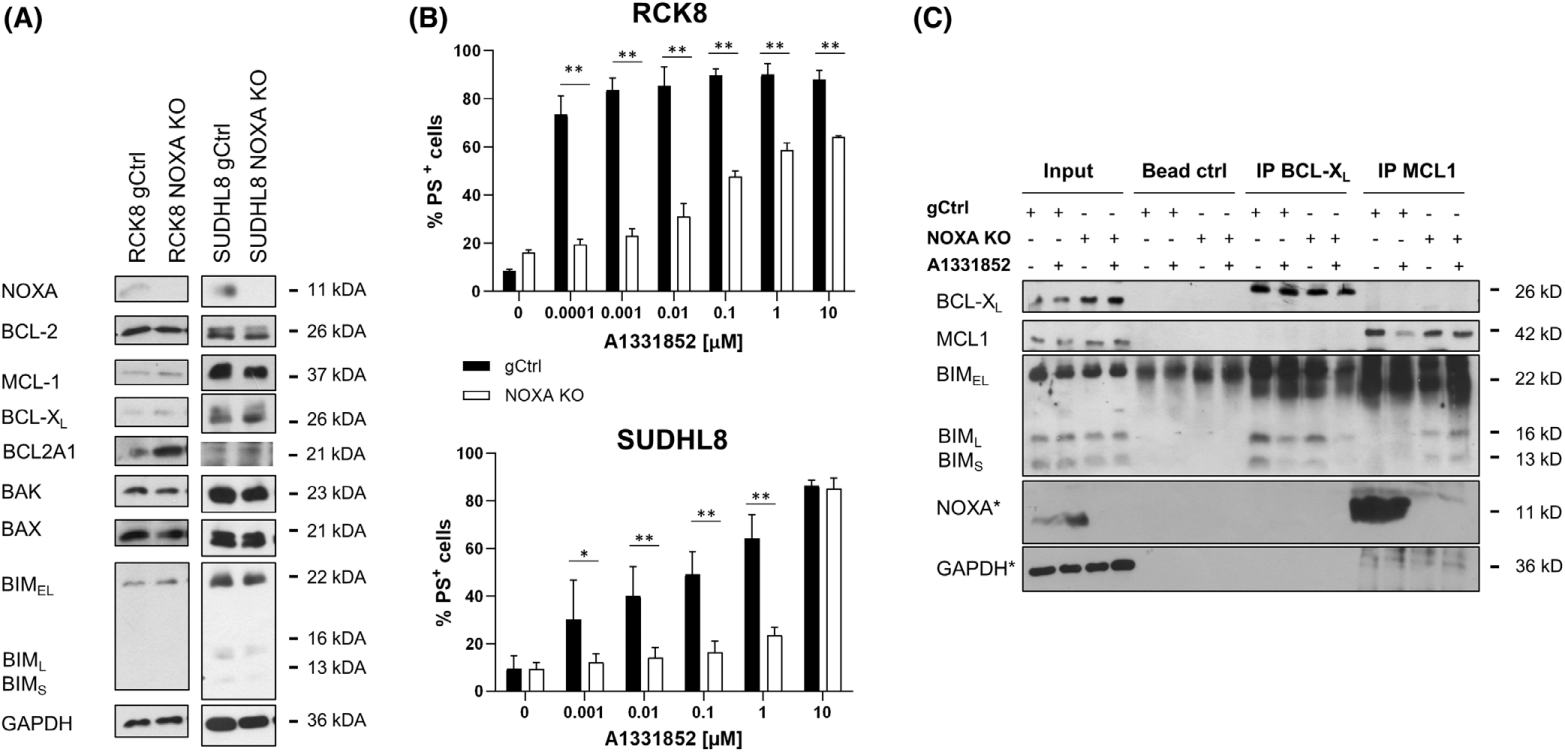
NOXA is essential for BCL‐X_L_ inhibitor‐induced apoptosis. (A) KO of NOXA was performed in RCK8 and SUDHL8 cells using CRISPR/Cas9. KO of NOXA and expression of BCL2 proteins in control (gCtrl) and NOXA KO cells was assessed by western blotting with GAPDH serving as loading controls. (B) Control and NOXA KO cells were exposed to different concentrations of A1331852 for 72 h before analysis of apoptosis using AnnexinV‐FITC and flow cytometry. Data presented are mean + SD (*n* > 3). *p < 0.05; **p < 0.01. (C) RCK8 control or NOXA KO cells were treated with A1331852 (3 nM) in the presence of zVAD.fmk (20 μM) for 6 h before immunoprecipitation of BCL‐X_L_ or MCL1. Binding of BIM, NOXA, and BAX was assessed by western blotting with GAPDH serving as loading control and (*) indicating individual blots required to detect all proteins.

**FIGURE 6:**
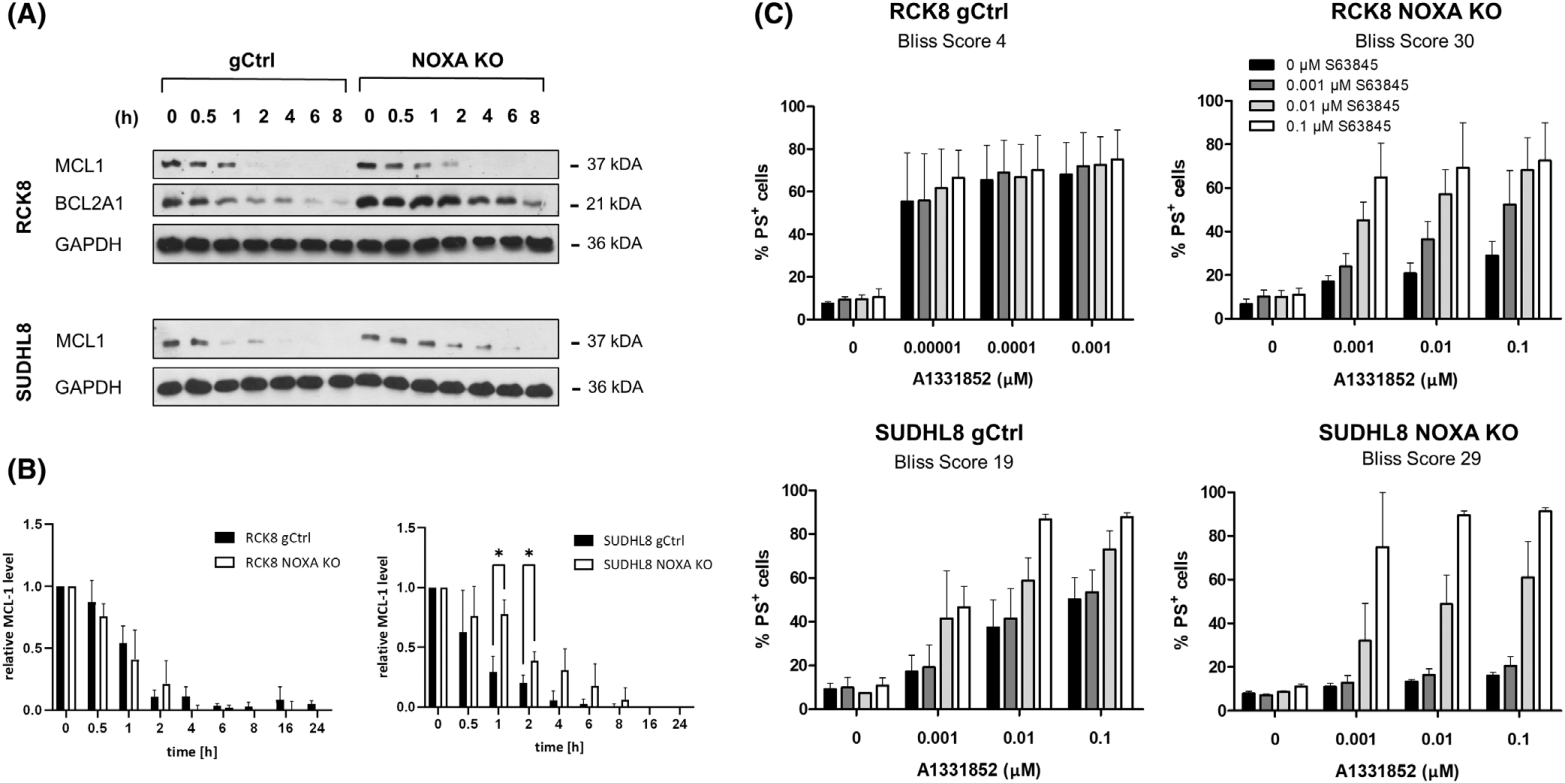
Loss of NOXA leads to increased MCL1 stability and function. (A, B) Protein stability was assessed in the RCK8 and SUDHL8 gCtrl or NOXA KO cells by treatment with cycloheximide (CHX, 100 μg/mL) for up to 8 h. Western blotting was performed with GAPDH serving as loading control. (B) Quantification of normalized MCL1 expression. *p < 0.05. (C) RCK8 and SUDHL8 control (gCtrl) or NOXA KO cells were treated with a combination of A1331852 and S63845 for 24 h before analysis of apoptosis using AnnexinV‐FITC and flow cytometry. Data presented are mean + SD (*n* > 3). Bliss score is indicated for the respective treatment.

Next, we asked whether NOXA may also be involved in apoptosis upon BCL‐X_L_ inhibition. Deletion of NOXA in the BCL‐X_L_‐dependent RCK8 and SUDHL8 cells^13^ did not result in expression changes within the BCL2 protein family except for the anti‐apoptotic protein BCL2A1, which was increased selectively in the RCK8 cells (Figure 5A). Deletion of NOXA resulted in a highly significant loss of sensitivity to A1331852 in both cell lines (Figure 5B) and caused reduced binding of BIM to BCL‐X_L_ and increased binding of BIM_L_ and BIM_S_ to MCL1, indicating that also here a shift of BIM to MCL1 was observed (Figure 5C). In line with the observations in the BCL2‐dependent cells, SUDHL8 NOXA KO cells displayed an increased protein stability of MCL1 (Figure 6A,B). In the RCK8 NOXA KO cells that displayed a higher expression of BCL2A1, its stability was increased, demonstrating that BCL2A1 may also be affected by the loss of NOXA. The combination of A1331852 with S63845 demonstrated higher synergy in the NOXA‐deleted cells compared to control cells, confirming an essential role of MCL1 in mediating the reduced sensitivity to the BCL‐X_L_ inhibitor in the absence of NOXA (Figure 6C). This was further supported by silencing of MCL1, which significantly increased the sensitivity to A1331852 in the RCK8 NOXA KO cells, whereas it had no effect in the RCK8 gCtrl cells (Figure S4).

Next, we investigated whether NOXA may also play a critical role in mediating sensitivity to the MCL1 inhibitor S63845. To this end, we deleted NOXA in the MCL1‐dependent cells U2946 and SUDHL10 (Figure S5). Also in these cells, in the absence of NOXA, more BIM_L_ and BIM_S_ were bound to MCL1 (Figure S5C). Interestingly, treatment with S63845 in the NOXA KO cells did not result in dissociation of BIM from MCL1 and, furthermore, sensitivity to S63845 was not altered. Taken together, this indicates that, as a direct binding partner of MCL1, NOXA may not be required for apoptosis induced by BH3‐mimetics targeting MCL1.

### Deletion of NOXA confers resistance to ABT‐199 in vivo

**FIGURE 7:**
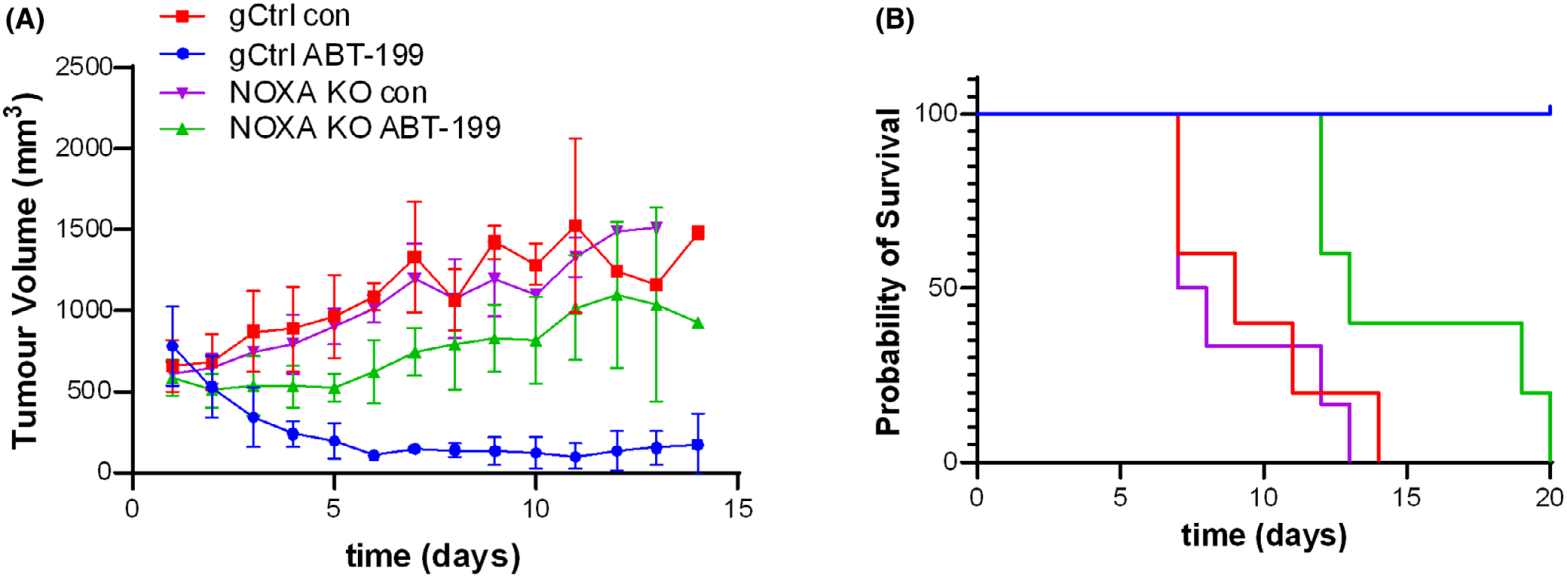
NOXA loss inhibits the response to ABT‐199 in vivo. (A, B) RIVA gCtrl or NOXA KO cells were transplanted subcutaneously into NSG mice. After tumour formation, mice were treated with ABT‐199 or vehicle for 3 days. Tumour volume was assessed by calliper (A) and survival of mice (B) was assessed. Data presented are mean + SD (*n* = 5).

Finally, we asked whether the effect of NOXA on ABT‐199‐induced cell death could also be observed in vivo. To this end, we performed xenograft experiments with the RIVA cells. In untreated control mice, tumour growth was comparable between the NOXA KO and gCtrl cells (Figure 7A). Treatment with ABT‐199 effectively reduced gCtrl tumour growth, whereas ABT‐199 only caused a slight delay in the growth of the NOXA‐deleted tumours. This was confirmed in the survival of the mice, where ABT‐199 prolonged the survival of the control mice, while all the NOXA KO mice succumbed to the disease within 20 days of treatment (Figure 7B). These data demonstrate that also in vivo NOXA plays an important role in the response to ABT‐199 treatment and that loss of NOXA was associated with ABT‐199 resistance. Taken together, these studies indicate that NOXA plays an essential role for apoptosis induced by either BCL2 or BCL‐X_L_ inhibition, which is at least partially mediated by MCL1 and may involve the interaction of BIM with MCL1.

## DISCUSSION

Here, we investigated the molecular mechanism associated with high apoptosis induction by ABT‐199 in DLBCL cells and identified the binding of BIM or NOXA to MCL1 as essential regulators of the response. While our studies concentrated on NOXA as a more selective binding partner of MCL1, a contribution of other BH3‐only proteins like PUMA or BMF cannot be excluded, and these may function in concert with BIM. Reduced NOXA expression, either by genetic deletion or in cells with intrinsically low NOXA expression, created free binding capacity and enabled MCL1 to also sequester BIM, in particular in situations where BIM was initially bound to BCL2 or BCL‐X_L_ but displaced by BH3‐mimetics. This confirms NOXA as a key binding partner of MCL1 and BIM as an important pro‐apoptotic signal able to shuttle between BCL2 and MCL1. This is in line with the ‘partner swapping’ model, where a BH3‐only protein displaced by BH3‐mimetics may now bind and neutralize another anti‐apoptotic protein like MCL1.^4^, ^27^ Our data indicate that while BIM may act as a versatile signalling molecule, the decision on its binding partners is dictated by NOXA and thus provides an explanation for the unique importance of NOXA in regulating the response to ABT‐199.

This model also implies a somewhat preferred binding of NOXA over BIM to MCL1, which may be explained by the higher binding affinity of NOXA over BIM. However, earlier studies investigating the binding of isolated BH3 domains did not show a higher affinity of NOXA but usually indicate tighter binding of BIM to MCL1.^28^, ^29^ Experiments performed on isolated BH3 domains may not accurately reflect the binding of proteins in cells, as other parts of the protein may influence binding.

Of note, we also find a similar molecular mechanism for BH3‐mimetics targeting BCL‐X_L_, whose ability to induce apoptosis is also highly influenced by NOXA. These data indicate a shared molecular mechanism whereby MCL1 neutralization via NOXA is required for efficient apoptosis induction following BCL2 or BCL‐X_L_ inhibition. In all of the DLBCL cells investigated, BIM was primarily bound to BCL2 or BCL‐X_L_ rather than MCL1.^13^ Interestingly, loss of NOXA had no effect on apoptosis induced by BH3‐mimetics targeting MCL1, showing that the displacement of NOXA from MCL1 alone does not induce ‘partner swapping’. This is supported both by the selective binding profile of NOXA as well as its short half‐life, indicating that displacement of NOXA from MCL1 may be non‐consequential for apoptosis induced by S63845. This is also supported by our findings that loss of NOXA per se does not alter apoptosis induced by the MCL1‐inhibitor S63845 in the BCL2 or BCL‐X_L_‐dependent cells. However, in the absence of NOXA, treatment with S63845 significantly increased apoptosis induced by either ABT‐199 or A1331852. Therefore, neutralization of MCL1 with S63845 may compensate for NOXA loss and enable ABT‐199 or A1331852 to induce apoptosis. Similarly, knockdown of MCL1 was per se not lethal in the NOXA KO cells but facilitated apoptosis induced by ABT‐199 or A1331852. Taken together, these data indicate that both BCL2/BCL‐X_L_ on the one side and MCL1 on the other side need to be neutralized for apoptosis induction.

In regard to future treatment options for DLBCL patients, our study has implications for protocols containing venetoclax like ViPOR.^30^ First, the basal NOXA expression may serve as a biomarker to stratify patients into venetoclax‐containing protocols. This is in line with studies highlighting the expression of anti‐ and pro‐apoptotic BCL2 proteins as well as their interaction patterns for the efficacy of BH3‐mimetics.^12^, ^13^, ^31^, ^32^ Second, durable responses to venetoclax may be more difficult to achieve in p53 dysfunctional malignancies.^33^, ^34^ With NOXA being a p53 target gene, in the absence of p53‐mediated gene expression NOXA may not be sufficiently expressed and thus limit the responses to venetoclax. Third, we and others have previously shown that proteasome inhibitors mainly sensitize DLBCL cells towards apoptosis by upregulating NOXA.^25^, ^35^ Improved clinical responses observed in the 5‐year follow‐up of DLBCL patients receiving bortezomib on top of R‐CHOP^36^ and in multiple myeloma patients with venetoclax and bortezomib^37^ support further investigations of similar combinations also in DLBCL patients.

In conclusion, our study demonstrates that the sensitivity to BCL2/BCL‐X_L_ inhibition is determined by the neutralization state of MCL1. In the absence of NOXA or chemical MCL1 inhibitors, MCL1 can sequester displaced BIM. However, in cells with NOXA expression, MCL1 is neutralized by NOXA, and the displaced BIM can activate apoptotic effectors BAK/BAX, highlighting NOXA as a mediator of sensitivity to BH3‐mimetics targeting BCL2 or BCL‐X_L_.

## AUTHOR CONTRIBUTIONS

N.Y., M.A., V.S., R.J.J, M.As. and S.J. performed experiments, generated and analysed data. M.J.S.D. and M.V. designed the study and analysed data. M.V. wrote the manuscript. All authors revised and approved the manuscript.

## FUNDING INFORMATION

This project was supported by the Else‐Kröner Fresenius Stiftung (to M.V.), the German Cancer Aid (to M.V.) and the Wilhelm Sander Stiftung (to M.V.) as well as the Scott Waudby Charitable Trust, to M.J.S.D.

## CONFLICT OF INTEREST STATEMENT

M.J.S.D. receives research funding from Beigene Pharma. All other authors declare no competing interest.

## Supporting information


Data S1.

## Data Availability

All primary data are available at the corresponding author upon reasonable request.
