# Supplementary material for: The BH3‐only protein NOXA is essential for apoptosis induction by BH3‐mimetics targeting BCL2 or BCL‐XL in DLBCL
Source: Br J Haematol. 2025 Oct 5;208(1):96–105. doi: 10.1111/bjh.70192 (PMC12819106; doi:10.1111/bjh.70192)
Supplement: Supplementary file 1 — Data S1. [file BJH-208-96-s001.pdf]

Supplemental Methods

Cell culture

The DLBCL cell lines DB, HBL-1, RIVA, U2932, WSU-DLCL2, SUDHL8 and RCK8 were derived from Deutsche Sammlung von Mikroorgansimen und Zellkulturen (DSMZ, Braunschweig, Germany). OCI-LY10 were kindly provided by Sandeep Dave, Duke University, Durham. Mycoplasma contamination testing was performed regularly. All cell lines were authenticated by short-tandem repeat (STR) profiling performed at DSMZ. For culture, Roswell Park Memorial Institute Medium (RPMI) 1640 GlutaMAX™-I (Life Technologies) was supplemented with 10% FCS (Biochrom) and 1% penicillin/streptomycin (Life Technologies). All cells were cultured at 37°C and 5% CO2 with saturated humidity and handled in a sterile environment under a laminar sterile hood.

Chemicals

Unless otherwise indicated, all chemicals were obtained from Sigma Aldrich. For induction of apoptosis, the BH3-mimetics ABT-199 (Selleck Chemicals), A1331852 (Selleck Chemicals) and S63845 (ApexBio) were used. To inhibit caspase activation, zVAD.fmk (Bachem) and to inhibit protein translation, cycloheximide (Sigma-Aldrich) were used. To induce NOXA expression, panobinostat (Selleck Chemicals), bortezomib (Selleck Chemicals), dBet6 (Tocris) or MLN4924 (Selleck Chemicals) were used.

CRISPR/Cas9 mediated gene knockout (KO)

To generate CRISPR/Cas9-mediated genetic KO cells, a two-vector system was used. First, the doxycycline inducible Cas9 containing plasmid, pCW-Cas9-Blast (Addgene #83481), was integrated into the cell line via lentiviral particles, and secondly, the pLentiguide-puro (Addgene #52963) was generated containing the following gRNA sequences: NOXA/PMAIP1 (CGCTCAACCGAGCCCCGCGC, TCGAGTGTGCTACTCAACTC and TTCTTGCGCGCCTTCTTCCC) and BIM/BCL2L11 (CTTGGGCGATCCATATCTCT, GTTCTGATGCAGCTTCCATG and TATGGATCGCCCAAGAGTTG). As non-targeting gCtrl

the following sequence was used: GCACTACCAGAGCTAACTCA. Viral particles were generated by transfection of HEK293T cells (ATCC) with the packaging constructs pMD2.g (Addgene #12259, 1 µg) and psPAX2 (Addgene #12260, 2.7 µg) using FugeneHD (Promega, Mannheim, Germany) and Optimem. The following day, the viral particle-containing supernatant was harvested and filtered through a 0.45 µm filter. DLBCL cells were seeded at a density of $2x10^6$ cells in 2 ml RPMI in 6-well plates supplemented with 4 µg/ml polybrene. The viral supernatant (0.5 ml/well) was directly added and the plate centrifuged for 2 h at 37°C and 1200 rcf. Cas9 was induced using doxycycline. Selection was performed using blasticidine (Sigma-Aldrich) and puromycin (Clontech).

siRNA mediated gene silencing

For transient silencing of genes, the following silencer select RNA sequences (ThermoFisher Scientific) were used: BCL-X_L/BCL2L1 (s1920, s1921) and MCL1 (s8383, s8385). Electroporation of $3x10^6$ DLBCL cells was performed using Neon® Transfection System (ThermoFisher Scientific) with 1200 V, 20 ms and 2 pulses. For efficient silencing, electroporation was repeated after 24 h. The cells were allowed to rest for following 2-4 h before start of experiments or control of knockdown by Western blotting.

Western blotting and IP experiments

For Western blotting, cells were lysed in TritonX100-containing buffer (30mM Tris-Base, 150mM NaCl, 1% Triton-X100, 10% Glycerol, 1× Protease Inhibitor Cocktail, 0.5mM PMSF, 2mM DTT, 1mM Sodium-orthovanadate, 1mM ß-glycerophosphate, 5mM Sodium-fluoride). Western blotting was performed using sodium dodecylsulfate polyacrylamide gel electrophoresis (SDS page) and semi-dry blotting onto a nitrocellulose membrane. For detection the following antibodies were used: anti-Caspase-3 (#9662S, Cell Signaling), anti-BCL2 (#M088701-2, Dako), anti-BCL-X_L (#2762S, Cell Signaling), anti-MCL1 (#ADI-AAP-240-F, Enzo), anti-MDR1 (#13342S, Cell Signaling), anti-PARP (#9542S, Cell Signaling), anti-NOXA (#ALX-804-408, Enzo), anti-BIM (#2819S, Cell Signaling), anti-PUMA (#4976S, Cell

Signaling), and BID (#2002S, Cell Signaling), anti-BAK (#06-536, Merck Millipore), anti-BAX (#2772S, Cell Signaling), anti-GAPDH (#5G4, HyTest), anti-Vinculin (#V9131, Sigma Aldrich). Visualization was done using Pierce TM ECL substrate according to manufacturer's instructions using X-ray films. The detection of activated BAX and BAK was evaluated using conformation-specific antibodies. Lysis was performed in CHAPS buffer (10 mM HEPES, 150 mM NaCl, 1% CHAPS, 1× Protease Inhibitor Cocktail, pH 7.4) before overnight incubation with 2 µl mouse anti-BAX (BAX clone 6A7, #B8429, Sigma Aldrich) or 2 µl mouse anti-BAK (BAK AB-1, #AM03, Calbiochem) antibodies and 10 µl pan mouse IgG dynabeads (#11042, Life Technologies). For interaction studies, hamster anti-BCL2 (#551051, BD Biosciences) or rabbit anti-MCL1 (#ADI-AAP-240-F, Enzo) antibodies were crosslinked to dynabeads™ Protein G (#10004D, Life Technologies) using 20 mM dimethyl pimelimidate (DMP, #21667, Sigma Aldrich).

Flow cytometry and viability assay

Annexin V conjugated with fluorescein isothiocyanate (FITC) was used to label phosphatidylserine (PS) on the surface of apoptotic cells with propidiumiodide (PI) addition. Cells were resuspended in Annexinbuffer (10 mM HEPES pH 7.4, 150 mM NaCl, 5 mM KCl, 1 mM $MgCl_2$, 1.8 mM $CaCl_2$) and flow cytometry was performed using FACSCanto (BD Biosciences). Cell viability was determined by CellTiter-Glo® Luminescent Cell Viability Assay (CTG, Promega) according to manufacturer's instructions.

Mass Spectronomy (MS)

For MS analysis, MCL1 IP was performed with anti-MCL1 antibody (Enzo) as described above using 3 mg of protein as input and dynabeads™ Protein G as control for unspecific binding. Immunprecipitates were washed in 50 mM Ammoniumcarbonat, dissolved in SDS loading buffer and separated on an SDS-page. Gel bands were sliced, and swollen in 50 mM Ammoniumcarbonat before destaining with acetonitrile. Trypsin was added to the gel slices for overnight digestion at 37 C. The peptides from the gel pieces were extracted into 0.2%

trifluoroacetic acid for 1 hour then aspirated and dried under reduced pressure. The dried samples were redissolved in injection solution and samples were submitted for Orbitrap MS performed using Q-Exactive (ThermoFisher). Analysis of interacting protein was done using PEAKS (Bioinformatics Solutions Inc, Canada) and visualized with Scaffold (Proteome Software, Oregon) using a 50% protein and 50% peptide threshold. To further eliminate unselectively binding proteins, all proteins were excluded that had in total over 2 replicates more than 10 peptide counts in the control beads.

In vivo experiments

Animal experiments conformed to the British Home Office Regulations (Animal Scientific Procedures Act 1986; Project Licences P8E5F4055 and PP9907621) and the guidelines for the welfare and use of animals in cancer research(17). This work was locally approved by the University of Leicester Animal Welfare Ethical Review Body sub-committee. RIVA gCtrl and RIVA NOXA KO cell suspension ($2.5x10^6$) prepared in 50:50 PBS:matrigel (Corning) were injected subcutaneously (max volume of 100 µl) in right dorsal flank into NOD scid gamma (NSG) mice. To measure the size of the tumor, a caliper was used. Once the average mean diameter/mean volume reached a size of 10 mm/500mm^3, the animals were blindly and randomly allocated in two treatment groups (vehicle vs ABT-199, n=5-6 per group). ABT-199 was formulated at 50 mg/kg (max 10 ml/kg) in 5% DMSO, 40% PEG-300, 5% Tween-80, 50% saline. Animals were treated with ABT-199 or vehicle by oral gavage, once per day, for 3 weeks, or until humane endpoints were reached.

Statistical analysis

Statistical analyses were performed using the GraphPad Prism software version 9. Statistical significance between two groups was tested by t-test, and more than two groups were determined by one-way ANOVA. P values less than 0.05 were considered statistically significant. Significance was marked as follows: *P<0.0.5, **P<0.01, ***P<0.001. Data points

not indicated are not significant. Drug interactions were analyzed via the Bliss independence model using the SynergyFinder web application (https://synergyfinder.fimm.fi/).

Supplementary Figure 1

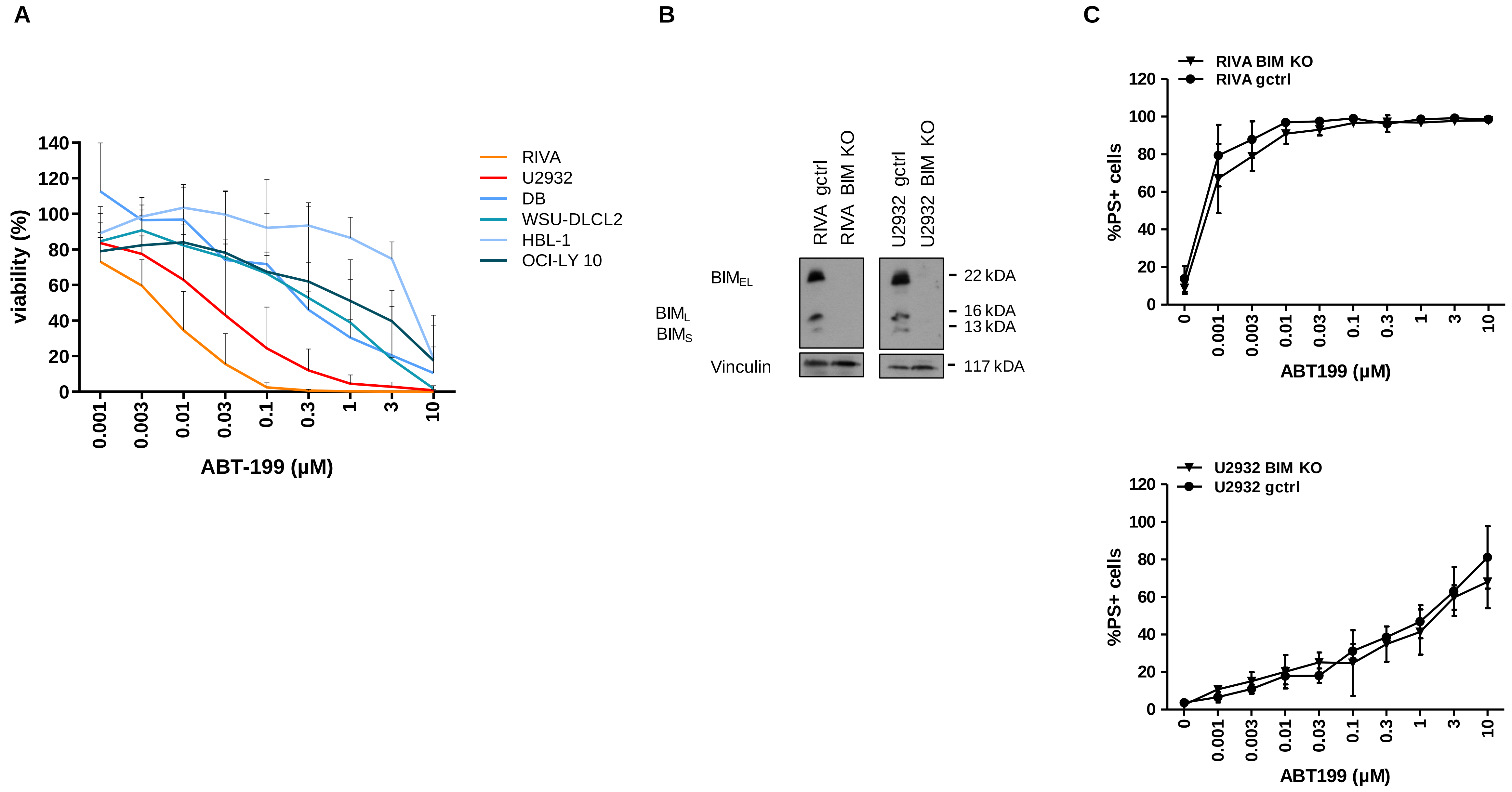

Supplementary Figure 2

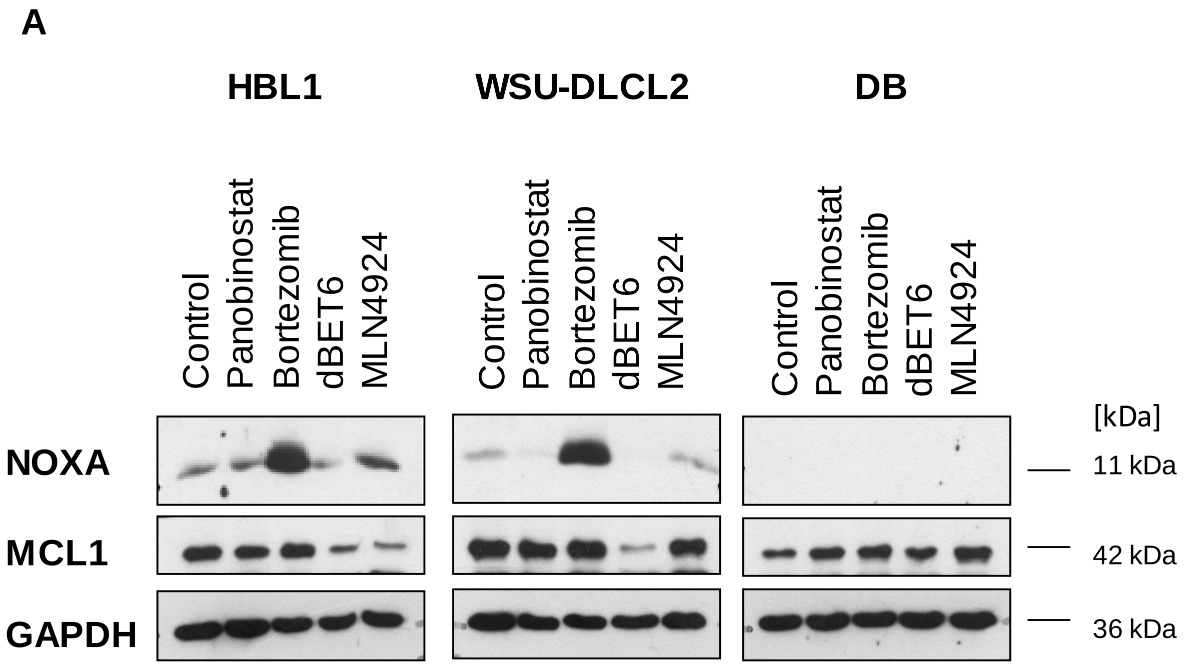

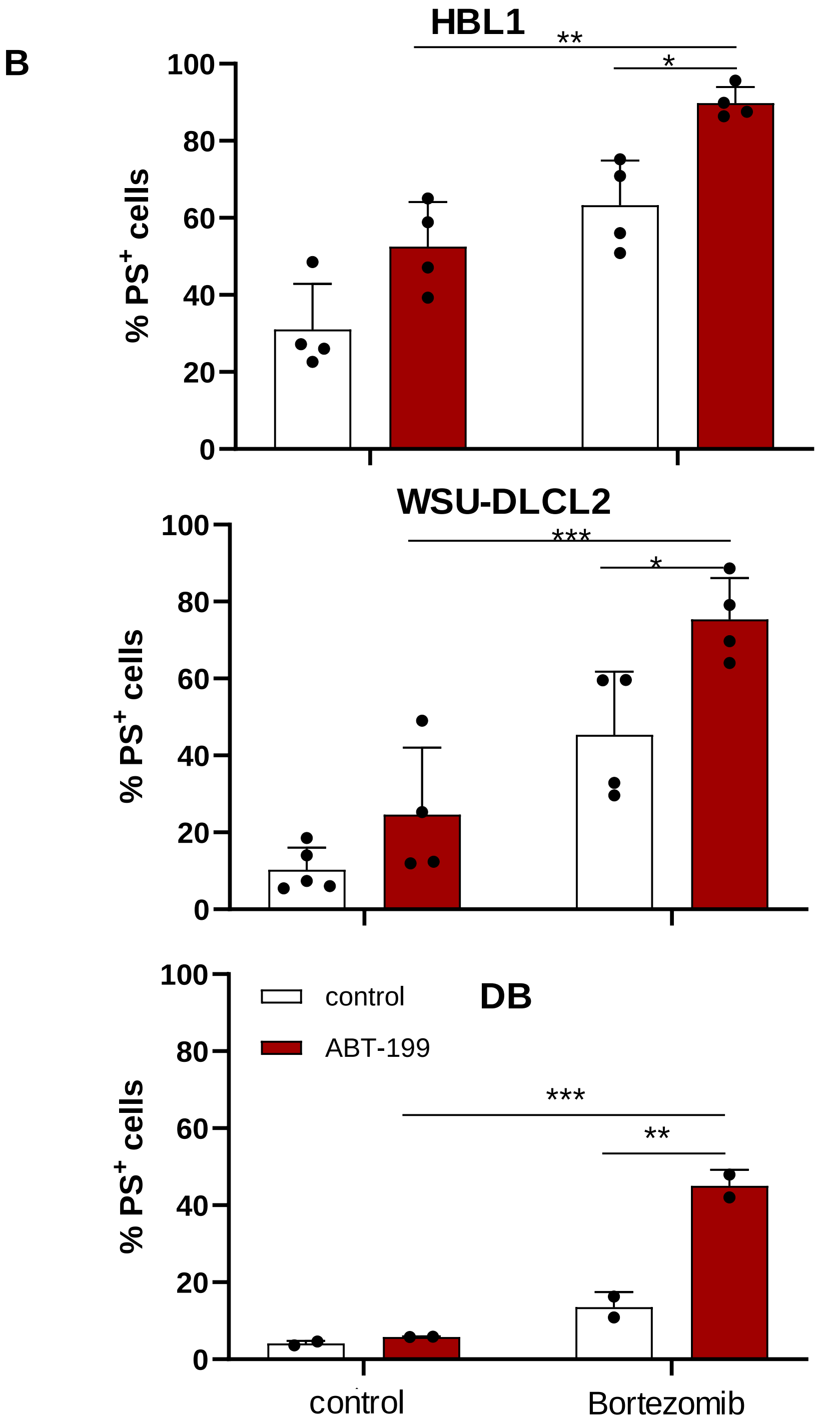

Supplementary Figure 3

	Beads only				MCL1 IP			
	gCtrl		NOXA KO		gCtrl		NOXA KO	
ABT-199	-	+	-	+	-	+	-	+
TRIM21	0	0	0	0	53	69	50	58
MCL1	0	0	0	0	50	34	23	26
DDX5	0	0	0	0	9	10	28	5
TPM3	0	0	0	0	4	12	22	9
TPM4	0	0	0	0	3	13	22	6
GCN1	0	0	0	0	1	9	21	2
PHB1	0	0	0	0	5	9	9	9
MCM7	0	0	0	0	4	10	16	1
RPL12	0	0	0	0	7	8	10	5
PRDX1	0	1	0	0	7	8	6	9
CAD	0	0	0	0	0	10	16	0
VDAC2	0	0	0	0	2	1	8	7
VDAC1	1	0	1	0	2	1	5	4
BCL2L11	0	0	0	0	0	0	1	10
MT-CO2	0	0	0	0	2	1	3	3
VDAC3	0	0	0	0	0	0	2	6
CDK1	0	0	0	0	2	2	4	0

Supplementary Figure 4

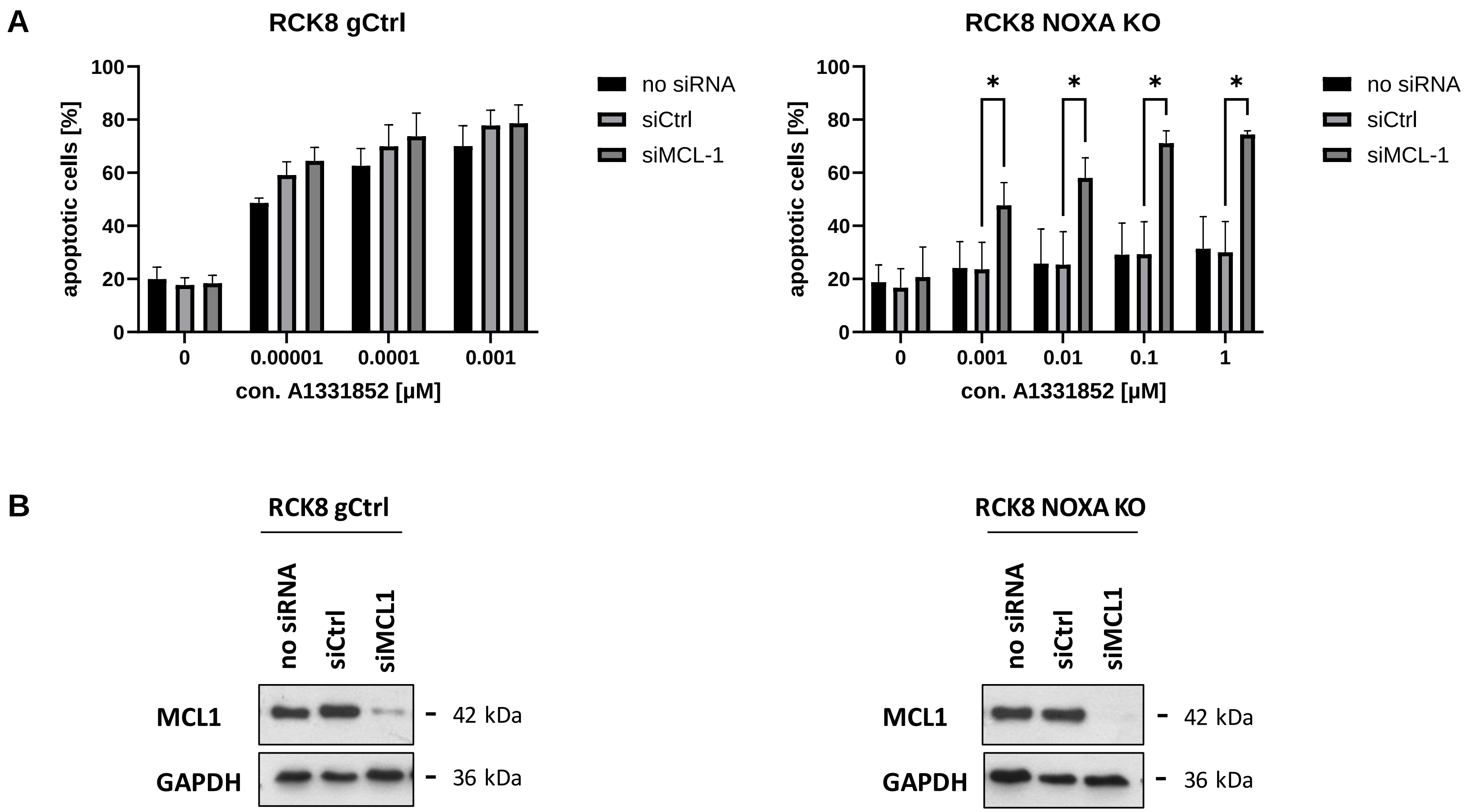

Supplementary Figure 5

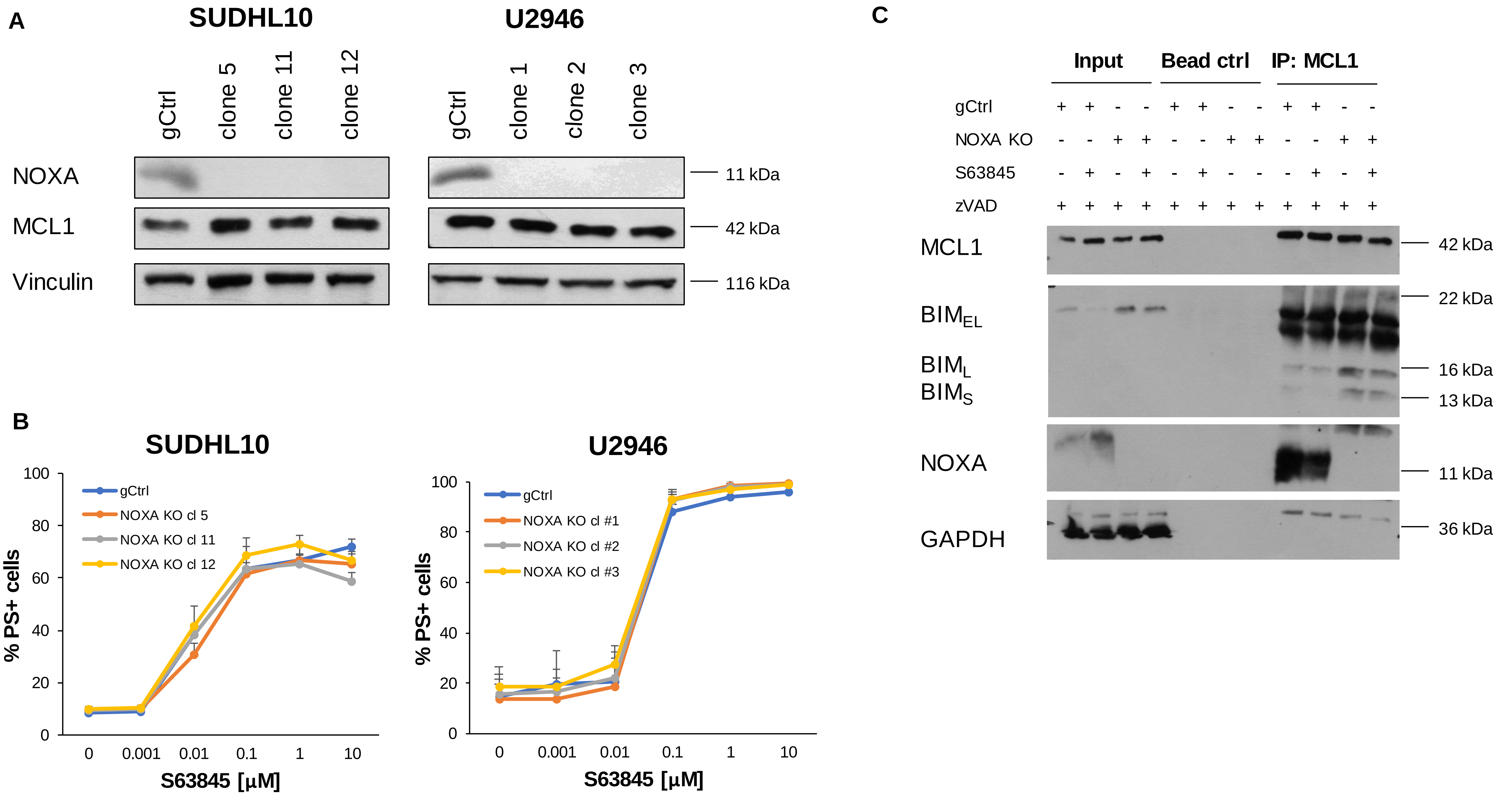

Supplementary Figure Legends

Supplementary Figure 1. Knockout of BIM has no effect on ABT-199-induced cell death

A) DLBCL cell lines were exposed to different concentrations of ABT-199 for 72 h before analysis of viability using CTG assay. Data presented are mean + S.D. (n>3). B-C) KO of BIM was performed in RIVA and U2932 cells using CRISPR/Cas9. B) Expression of BIM in control (gCtrl) and BIM KO cells was assessed by Western blotting with Vinculin serving as loading control. C) Control and BIM KO cells were exposed to different concentrations of ABT-199 for 72 h before analysis of cell viability using CTG assay. Data presented are mean + S.D. (n>3).

Supplementary Figure 2. Induction of NOXA to synergize with ABT-199

A) HBL1, WSU-DLCL2 and DB cells were exposed to panobinostat (0.01 μM), bortezomib (0.01 μM), dBET6 (0.1 μM) or MLN4924 (10 μM) for 24h before analysis of protein expression by Western botting. B) Cells were treated with bortezomib (0.01 μM) and ABT-199 (1 μM) for 24h before analysis of apoptosis using AnnexinV-FITC and flow cytometry. Data presented are mean + S.D. (n>3). **P* <0.05; ***P* <0.01; ****P* <0.001.

Supplementary Figure 3. Lis of potential binding partners of MCL1

MS analysis of MCL1 IP and Beads only control performed in RIVA cells. RIVA control (gCtrl) or NOXA KO cells were treated with ABT-199 (0.1 μM) in the presence of zVAD.fmk (20 μM) for 6 h before immunoprecipitation of MCL1. Selected interaction partners are displayed in a table with their indicated peptide counts and colour grading.

Supplementary Figure 4. Knockdown of MCL1 in RCK8 cells

A) Transient silencing of MCL1 was performed in RCK8 control (gCtrl) or NOXA KO cells before treatment of cells with different concentrations of A1331852 for 24 h and analysis of apoptosis using AnnexinV-FITC and flow cytometry. Non-transfected (no siRNA) or non-targeting siRNA (siCtrl) were used as controls. Data presented are mean + S.D. (n>3). **P* <0.05. B) Knockdown efficacy was assessed by Western blotting with GAPDH serving as loading control.

Supplementary Figure 5. Knockout of NOXA has no effect on S63845-induced cell death

A) KO of NOXA was performed in SUDHL10 and U2946 cells using CRISPR/Cas9. Expression of NOXA in control (gCtrl) and different KO clones was assessed by Western blotting with Vinculin serving as loading control. B) Control and NOXA KO cells were exposed to different concentrations of S63845 for 24 h before analysis of cell death by staining with AnnexinV-FITC and detection of phosphatidylserine (PS) exposure using flow cytometry. Data presented are mean + S.D. (n=3). C) U2946 gCtrl or NOXA KO cells were left untreated or exposed to S63845 (0.1 μM) and zVAD.fmk (20 μM) for 6h before lysis and immunoprecipitation. Immunoprecipitation of MCL1 was analyzed for binding of BIM and NOXA using Western blotting with Lysate serving as Input and Bead ctrl as control for unspecific binding.
